# Developmental Dental Aberrations After the Dioxin Accident in Seveso

**DOI:** 10.1289/ehp.6920

**Published:** 2004-07-01

**Authors:** Satu Alaluusua, Pier Calderara, Pier Mario Gerthoux, Pirjo-Liisa Lukinmaa, Outi Kovero, Larry Needham, Donald G. Patterson, Jouko Tuomisto, Paolo Mocarelli

**Affiliations:** ^1^Department of Pedodontics and Orthodontics, Institute of Dentistry, University of Helsinki, Helsinki, Finland; ^2^Department of Oral and Maxillofacial Diseases, Helsinki University Central Hospital, Helsinki, Finland; ^3^Department of Laboratory Medicine, Desio Hospital, University of Milano, Bicocca, Italy; ^4^Department of Oral Pathology, Institute of Dentistry, University of Helsinki, and Department of Pathology, Helsinki University Central Hospital, Helsinki, Finland; ^5^Department of Radiology, Institute of Dentistry, University of Helsinki, Helsinki, Finland; ^6^Division of Laboratory Sciences, Centers for Disease Control and Prevention, Atlanta, Georgia, USA; ^7^Department of Environmental Health, National Public Health Institute, and Department of Public Health and General Practice, University of Kuopio, Kuopio, Finland

**Keywords:** developmental enamel defect, dioxin, hypodontia, hypomineralization, hypoplasia, Seveso, TCDD, teeth

## Abstract

Children’s developing teeth may be sensitive to environmental dioxins, and in animal studies developing teeth are one of the most sensitive targets of toxicity of 2,3,7,8-tetrachlorodibenzo-*p*-dioxin (TCDD). Twenty-five years after the dioxin accident in Seveso, Italy, 48 subjects from the contaminated areas (zones A and B) and in patches lightly contaminated (zone R) were recruited for the examination of dental and oral aberrations. Subjects were randomly invited from those exposed in their childhood and for whom frozen serum samples were available. The subjects were frequency matched with 65 subjects from the surrounding non-ABR zone for age, sex, and education. Concentrations of TCDD in previously analyzed plasma samples (zone ABR subjects only) ranged from 23 to 26,000 ng/kg in serum lipid. Ninety-three percent (25 of 27) of the subjects who had developmental enamel defects had been < 5 years of age at the time of the accident. The prevalence of defects in this age group was 42% (15 of 36) in zone ABR subjects and 26% (10 of 39) in zone non-ABR subjects, correlating with serum TCDD levels (*p* = 0.016). Hypodontia was seen in 12.5% (6 of 48) and 4.6% (3 of 65) of the zone ABR and non-ABR subjects, respectively, also correlating with serum TCDD level (*p* = 0.05). In conclusion, developmental dental aberrations were associated with childhood exposure to TCDD. In contrast, dental caries and periodontal disease, both infectious in nature, and oral pigmentation and salivary flow rate were not related to the exposure. The results support our hypothesis that dioxins can interfere with human organogenesis.

2,3,7,8-Tetrachlorodibenzo-*p*-dioxin (TCDD) is referred to as the most toxic man-made chemical known [International Agency for Research on Cancer (IARC) 1997]. Data on environmental accidents and occupationally exposed subjects have increased our knowledge on human health effects of TCDD (Sweeney and Mocarelli 2000).

The best-known dioxin accident took place in Seveso, Italy, in 1976. In a chemical factory, a trichlorophenol production reactor exploded and ≥30 kg TCDD was spread over an area of approximately 18 km^2^ (Di Domenico et al. 1980). Several thousand people, children among them, were exposed to substantial quantities of TCDD. The contaminated area was divided into three major zones (A, B, and R) on the basis of decreasing order of surface soil concentrations of TCDD (Bisanti et al. 1980), and soon after the accident a health assessment of the population was initiated. As part of this effort, blood samples were collected for clinical chemistry tests, and small amounts of serum were stored for later analyses. These samples rendered it possible to quantify individual TCDD exposures (Mocarelli et al. 1988; Needham et al. 1997/1998).

Children exposed to TCDD had higher body burdens than adults. This was seen in zones A and B but also in the non-ABR zone surrounding the three major zones (Eskenazi et al. 2004). Approximately 20% of the children < 10 years of age who had been exposed and had been living in the most severely contaminated zone A developed chloracne (Mocarelli et al. 1986). Exposure of males before and during puberty was linked to a lower male:female ratio in their offspring, and it was suggested that TCDD permanently affected the function of human epididymis (Mocarelli et al. 2000).

In two episodes of epidemic poisoning in Japan and Taiwan (so-called Yusho and Yucheng accidents, respectively), severe developmental effects were observed in infants and children born to mothers who had been exposed to polychlorinated dibenzofurans/biphenyls (PCDFs/PCBs) (Rogan et al. 1988; Yamashita and Hayashi 1985; Yao et al. 2002). These included intrauterine growth retardation, low birth weight, hyperpigmentation, natal teeth, increased incidences of skin and respiratory infections, neurodevelopmental delay, and alterations in sexual development (Chen and Hsu 1994; Chen et al. 1992; Ikeda 1996; Rogan et al 1988).

Although there are differences among species in susceptibility, embryonic development of most vertebrate species is sensitive to TCDD (Peterson et al. 1993). Developmental effects in laboratory rodents include cleft palate, disturbed development of the mandible, various ureteric and kidney malformations in mice (Abbott and Birnbaum 1989; Abbott et al. 1987; Allen and Leam 2001; Peters et al. 1999), and alterations in the reproductive tract development and function in mice and rats (Hurst et al. 2000; Theobald and Peterson 1997). Impaired mammary gland development and differentiation were found in female mice after gestational and lactational exposure (Lewis et al. 2001). In rhesus macaques TCDD causes developmental jaw cysts (McNulty 1985), and in avian and fish embryos it causes cardiovascular toxicity and disturbs craniofacial development (Cantrell et al. 1996; Hornung et al. 1999; Teraoka et al. 2002; Walker and Catron 2000).

Teeth develop as a result of a series of inductive, sequential, and reciprocal interactions between the ectoderm and the subjacent mesenchyme (Thesleff et al. 1995). Tooth development is genetically regulated but sensitive to environmental disturbances. Aberrations in the function of tooth-forming cells lead to permanent morphologic consequences. Because development of the first primary tooth begins in the fourth week *in utero* and the development of the roots of the wisdom teeth is completed around 20 years of age, teeth serve as a record that covers a long time period of life.

We have shown that in a normal child population, polychlorinated dibenzo-*p*-dioxins (PCDDs) and PCDFs in mother’s milk may cause mineralization defects in the child’s permanent first molar teeth undergoing mineralization during the first 2 years of life (Alaluusua et al. 1996, 1999). A variety of dental and oral changes were also reported in children exposed to PCB/PCDF in the Yusho and Yucheng accidents. At birth natal teeth and oral pigmentation were prevalent, and later, missing permanent teeth, delayed eruption of permanent teeth, and disturbed root development were observed (Akamine et al. 1985; Fukuyama et al. 1979; Rogan et al. 1988). In adulthood, periodontal disease was common (Hashiguchi et al. 2003). However, in these studies, individual serum or human milk levels of the contaminants in the exposed subjects were not available.

Previous studies *in vivo* and *in vitro* show that rat and mouse teeth are sensitive to TCDD throughout their development (Alaluusua et al. 1993; Kattainen et al. 2001; Kiukkonen et al. 2002; Lukinmaa et al. 2001; Miettinen et al. 2002; Partanen et al. 1998). The dental toxicity of TCDD appears to have two phases (Partanen et al. 1998). First, the sequence of morphogenetic events can be affected. This leads to failure of tooth germs to develop as a result of accelerated and increased apoptosis in the dental lamina connecting the oral epithelium and the tooth germ (Lukinmaa et al. 2001; Partanen et al. 2004). Later, tooth size can be reduced (Kattainen et al. 2001; Miettinen et al. 2002; Partanen et al. 1998, 2004). In more advanced teeth, root development can be arrested (Lukinmaa et al. 2001). Second, the interference by TCDD with the formative stage of tooth development—that is, the function of secretory ameloblasts and odontoblasts—results in delayed or defective mineralization of the molar teeth (Gao et al. 2004; Partanen et al. 1998) and failure of enamel and dentin formation to be completed in the continuously erupting rat incisors (Alaluusua et al. 1993; Kiukkonen et al. 2002; Lukinmaa et al. 2001).

In rodents, effective doses of TCDD are very low; for example, a single dose of 30 ng/kg to the rat dam during gestation reduced molar tooth size in the pup (Kattainen et al. 2001). That dose produces maternal concentrations in adipose tissue that are not markedly different from those at the high end of human adipose tissue concentrations without occupational or accidental exposure and is one to three orders of magnitude lower than the serum lipid concentrations measured in subjects after accidental exposure (Hurst et al. 2000; Needham et al. 1999).

Because of all these facts, 25 years after the Seveso accident we invited subjects exposed to TCDD in 1976 in their childhood to receive a dental examination. The goal was to determine the dental and oral changes after heavy exposure to TCDD. We hypothesized that TCDD causes developmental dental defects that can still be seen in adulthood. Furthermore, we expected to see increased prevalences of periodontal disease and oral pigmentation among the exposed subjects, as found in the Yusho subjects (Hashiguchi et al. 2003). We have found that TCDD causes morphologic changes in cultured mouse embryonic salivary glands (Kiukkonen A, et al., unpublished data). Therefore, we also wanted to find out whether salivary flow rate is normal in the exposed subjects.

## Materials and Methods

Sixty-five subjects from the contaminated A and B zones and from the R zone, which was lightly contaminated in patches, and 130 subjects from the surrounding, so-called non-ABR zone were invited for the study. At the time of the accident, the subjects were < 9.5 years of age. Demographic information was officially obtained through the different municipalities’ censuses and consisted of date of birth and town of residence in July 1976. The subjects from ABR zones were randomly selected among those for whom we had frozen serum samples since 1976. TCDD in serum had been previously measured by high-resolution gas chromatography/isotope-dilution high-resolution mass spectrometry (Needham et al. 1999; Patterson et al. 1987). Serum samples from non-ABR zone subjects were not available. The eligible subjects were contacted by letter and by phone by the same person. The compliance for the zone ABR subjects and non-ABR subjects was 74 and 58%, respectively. Written informed consent was obtained and approval given by the local institutional review board.

A structured questionnaire, which included a collection of a detailed personal dental and medical history, education, and smoking habits, was administered to all subjects through personal interview. The subjects were frequency matched for age, sex, and education. Education, which is known to modify dental health, was categorized into five levels. Elementary school and secondary school not finished were scored as “lower education”; vocational school, secondary school finished, and high school finished, with or without university training, were scored as “higher education” (Table 1). Because data obtained by the questionnaire showed that the non-ABR group had a higher education level, we randomly dropped out 10 subjects who had finished high school or had university training. Thus, the final number of subjects from the ABR zones was 48 and from the non-ABR zone was 65.

One dentist (P.C.) undertook the examination in a dental unit kindly provided by Planmeca Oy (Helsinki, Finland). During the examination, the dentist had no knowledge whether the subject had been an ABR or non-ABR resident or of the serum TCDD concentration. The subjects were not aware of the hypothesis under study. All teeth (excluding wisdom teeth) were recorded for agenesis of tooth and developmental dental defects using the Developmental Defects of Enamel Index (Fédération Dentaire Internationale 1992). The lesions were grouped into three types: demarcated opacities and diffuse opacities (both of which are qualitative defects of enamel) and hypoplasia (quantitative defect of enamel). Defects involving a local alteration in the translucency of the enamel with clear boundary to the adjacent normal enamel were recorded as demarcated opacities. Diffuse type of opacities included defects involving an alteration in the translucency of the enamel with no clear boundary to the adjacent normal enamel. Hypoplasia included defects with reduced thickness of enamel, the morphology of the defects ranging from shallow or deep pits, small or large, wide or narrow grooves to partial or complete absence of enamel over small or considerable areas of dentine. Lesions < 2 mm in diameter and hereditary defects in tooth structure or tetracycline staining were not included in the analysis. To evaluate the intra-examiner reproducibility of the investigator, 25 subjects were reevaluated a few weeks later and results were assessed using Cohen’s κ-coefficient (Pine et al. 1997). Reproducibility on the presence of defects in a subject was 100%. The κ-statistic on tooth basis was 0.73.

Caries was assessed according to recommendations of the World Health Organization (WHO 1997). Periodontal status was determined by examining six surfaces of all teeth (midbuccally, midlingually, and approximally both buccally and lingually) for the presence or absence of gingival bleeding on probing, subgingival calculus, and pocket depth as assessed by a ball point probe graded in 2 mm (probe force ~ 20 g; LM Instruments Oy, Parainen, Finland). Gingiva, palate, buccal mucosa, and tongue were examined for pigmentation. For the measurement of salivary flow, paraffin-stimulated saliva was collected for 5 min.

The clinical examination was supplemented by radiographic examination using panoramic tomography. Missing or retention of teeth, alveolar bone loss, root deformities, and the presence of jaw cysts were recorded. One dentist (O.K.) undertook the radiographic examinations, and during the examination she had no knowledge whether the subject belonged to the control or the study group or of the serum TCDD concentration.

Pearson’s correlations were computed on the entire study population and for both sexes separately to examine associations between different variables. For analysis of associations between serum TCDD concentrations and other variables, residents from ABR zones were divided into three groups, in an increasing order of serum TCDD concentrations. Each group included 16 subjects. For studying the role of age on the prevalence of developmental enamel defects, the study population was divided into two groups: subjects < 5 years of age and subjects > 5 years of age at the time of the accident. This grouping was based on the fact that enamel development in the permanent dentition (excluding wisdom teeth) is most sensitive to environmental disturbances up to the first 5–7 years of life. Cumulative odds ratio for developmental enamel defects was calculated on ranked serum TCDD concentrations for the < 5 year age group. Logistic regression, where the presence of developmental defects of enamel was a response variable and education (two levels) and serum TCDD concentration (three levels) were explanatory variables, was calculated. Corresponding odds ratios with their 95% confidence intervals were determined. Differences between the means were evaluated by independent-sample *t*-test or by Mann-Whitney *U*-test. Comparisons between the categorized variables were done by chi-square test and Mantel-Haenszel chi-square test. In all statistical tests, probabilities ≤0.05 were considered statistically significant.

## Results

At the time of the dental and oral examination, the subjects from ABR zones were 25.4–34.0 years of age (mean, 29.1), and those from the non-ABR zone were 24.6–34.1 years of age (mean, 29.2). None of them had experienced severe disease such as cancer in their childhood.

Characteristics of the study population are shown in Table 1. Like sex, age, and education level, smoking habits were not significantly different in zone ABR and non-ABR residents. TCDD concentrations of the zone ABR subjects ranged from 23 to 26,000 ng/kg (median, 476 ng/kg) in serum lipid. At the lowest tertile, TCDD values ranged from 23 to 226 ng/kg in serum lipid, at the mid-tertile from 238 to 592, and at the highest from 700 to 26,000, respectively.

### Developmental defects of enamel.

**Figure 1:**
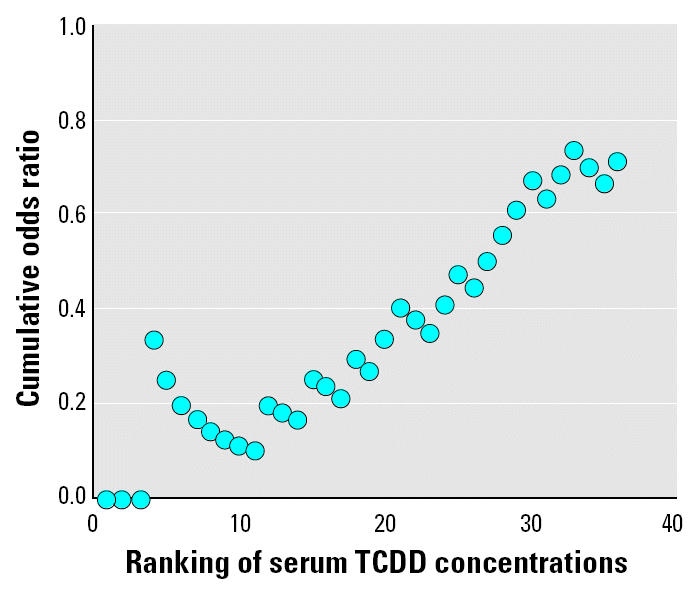
Ratio of subjects with developmental dental defects to those without at each exposure level. Subjects < 5 years of age at the time of the Seveso accident and from whom serum samples were collected soon after the accident were included (*n* = 36). Range of the TCDD concentrations was 31–26,000 ng/kg in serum lipid.

Developmental defects of enamel were found in 27 subjects (14 males, 13 females). All but two of them had been < 5 years of age at the time of the accident (*p* < 0.0009; Table 2). The prevalence of defects in the subjects from the ABR zones was 42% (15 of 36) and that from non-ABR zone was 26% (10 of 39; Table 2). Subjects with higher serum TCDD levels had more frequent developmental defects of enamel than did those with lower TCDD levels (Mantel-Haenszel χ^2^ = 5.76, *p* = 0.016; χ^2^ = 6.26, *p* = 0.044; Tables 2 and 3), and at ranked serum concentration levels, the ratio of subjects with developmental defects of enamel to those without such defects increased (Figure 1). In the older age group, the prevalence of defects was only 5.3% (2 of 38).

Education level was negatively associated with the presence of developmental enamel defects in subjects who had been < 5 years of age at the time of the accident (χ^2^ = 7.14, *p* < 0.0075; Tables 2 and 3). The association was clearer among the zone ABR subjects (χ^2^ = 5.14, *p* = 0.023) than in the zone non-ABR subjects (χ^2^ = 2.21, *p* = 0.14). Logistic model revealed no significant interaction between low educational level and the level of serum TCDD concentration.

The difference in the prevalence of developmental defects of enamel between zone ABR and non-ABR subjects who had been < 5 years of age at the time of the accident was due to the high number of teeth with enamel hypoplasia in the zone ABR subjects. Seven of the zone ABR subjects (19.4%) had at least one hypoplastic tooth compared with two subjects from the non-ABR zone (5.1%). Demarcated opacities occurred in 8 of 36 zone ABR subjects (22.2%) and in 10 of 39 zone non-ABR subjects (25.6%). Two subjects had both demarcated opacities and hypoplasia. Diffuse opacities related to fluorosis were not seen.

### Hypodontia.

A total of 12.5% of the zone ABR subjects (three males, three females) had missing permanent teeth (excluding wisdom teeth), compared with 4.6% of the zone non-ABR residents (two males, one female). The teeth missing were lateral incisors and second premolars. Zone ABR subjects with higher serum TCDD levels more often lacked permanent teeth than did those with lower TCDD levels or the zone non-ABR residents (Mantel-Haenszel χ^2^ = 3.83, *p* = 0.05).

### Other dental and oral aberrations.

Other pathologic changes in the oral cavity that could be related to the exposure to TCDD were few. Dilaceration of tooth roots was not seen. Two zone ABR and two zone non-ABR subjects had gingival pigmentation. No significant associations between any periodontal parameters and serum TCDD levels were seen, and the number of deepened periodontal pockets, percentage of subgingival calculus sites, and percentage of bleeding sites after probing were similar in subjects from zones ABR and non-ABR (Table 4). Prevalence of caries and salivary flow rate were also on similar levels in both groups (Table 4). Significant associations between caries or salivary flow rate and serum TCDD concentrations were not found.

## Discussion

Twenty-five years after the Seveso accident we found that serum TCDD levels in childhood were associated with the presence of developmental enamel defects in the permanent dentition. This study, supporting our earlier results of the sensitivity of developing teeth to dioxins, was possible to perform because of two important conditions. First, serum samples from children after the accident had been collected and stored for further analysis. Second, tracing of developmental defects of enamel after such a long time was possible because teeth, once they have developed, do not undergo remodeling; that is, the defects are permanent in nature.

Ninety-three percent of the subjects with enamel defects had been < 5 years of age at the time of the accident. Enamel development of the permanent dentition (excluding wisdom teeth) is most sensitive to environmental disturbances up to the first 5–7 years of life, when mineralization of the crowns is radiographically completed (Haavikko 1970). Because of the vulnerability of the forming enamel, the distribution pattern supports the role of TCDD as causative of the defects.

Among both zone ABR and non-ABR subjects, developmental enamel defects were more prevalent in those subjects who were < 5 years of age at the time of the accident than in those who were older. A recent study by Eskenazi et al. (2004) showed that in two pooled samples from 40 children (0–12 years of age) who lived in the non-ABR zone in 1976, serum contained 33.4 and 47.6 ng/kg TCDD in lipid. These concentrations overlap those measured from children who lived in the ABR zones. Effects of TCDD and other dioxin-like compounds (also found in the pooled samples) on prevalence figures of developmental defects of enamel (26% in zone non-ABR subjects vs. 42% in zone ABR subjects) on these “background” levels cannot be excluded.

Likewise, in an epidemiologic study on 8-to 15-year-old children pre- and postnatally exposed to polychlorinated aromatic hydrocarbons, mainly PCBs, Jan and Vrbič (2000) found that significantly more developmental defects of enamel were found in a contaminated region than in the control area. This suggests that exposure to PCBs can also be associated with developmental enamel defects. However, whether dioxins and PCBs share the mechanism(s) of interference with tooth development is not known.

The subjects from the ABR and non-ABR zones were frequency matched with age, sex, and education. Developmental defects of enamel are persistent but can be masked by caries and can be removed in connection of treatment of caries (e.g., fillings, crowns). Education and age are related to dental health (Brodeur et al. 2000; Reisine and Psoter 2001). To exclude the possible bias caused by difference in education level between the groups, we randomly dropped 10 subjects initially included in the non-ABR group. The DMFT (number of decayed, missing, and filled teeth due to caries) index, which tells the number of teeth affected by caries, was 11.7 in the subjects from ABR zones and 10.6 in the subjects from the non-ABR zone, indicating that “masking” of developmental defects was at a similar level in the dentitions of both groups.

We found that education was negatively correlated with the presence of developmental defects of enamel in subjects who at the time of the accident were < 5 years of age, but not in the older age group. In the younger age group 8 of 12 subjects with basic education only had developmental defects of enamel, whereas in the older age group the proportion was 1 of 8. The difference in the distribution is difficult to explain. We therefore studied whether an interaction between the education level and the serum TCDD level could explain the higher prevalence of developmental dental defects in subjects with basic education only, but such an interaction was not found. Education thus remained as an independent explanatory factor.

Here we evaluated the association between the exposure to TCDD and the presence of developmental dental defects. Earlier, almost 100 different factors had been listed as being responsible for developmental defects of enamel (Small and Murray 1978). Therefore, it is more than likely that many defects seen in the study population were not related to exposure to TCDD. Hypoplastic enamel defects were found here in higher numbers than in many other populations (Fédération Dentaire Internationale 1992; Wiktorsson et al. 1994). However, the same etiologic factor may cause enamel opacity and hypoplasia, the end result depending on timing, duration, and severity of the influence of the disturbing agent and the susceptibility of the individual. Therefore, here, as well as in other conditions, it is rarely possible to connect a clinical appearance of a defect with a particular causative agent.

Mineralization of the first permanent teeth starts around birth and of the last usually between years 2 and 3 of life, although the normal range is wide (Schour and Massler 1940). Before mineralization, severe damage such as mechanical trauma to the tooth germ as well as multiagent chemotherapy and radiation therapy may arrest the development, leading to absence of a tooth. However, in most cases the basis for a missing tooth is genetic. Prevalence figures for nonsyndromic hypodontia (one to six teeth missing) in the permanent dentition in a normal population differ to some extent among countries, but most studies show prevalences of 3–8% (Arte 2002). Childhood exposure to PCBs and PCDFs may have increased the prevalence of hypodontia in Yusho subjects. Accordingly, 3 of 37 children (9%) had missing teeth (Fukuyama et al. 1979). In our study the prevalence was 3 of 16 (19%) in subjects with the highest tertile of serum TCDD concentrations, compared with 3 of 65 (4.6%) in the subjects from the non-ABR zone. The result of an increased prevalence of hypodontia in our study is in line with the studies in Yusho and our observations in laboratory rodents, but should be interpreted with caution because of the small study population and small number of subjects with hypodontia. To confirm that TCDD is related to hypodontia, a larger study population of children < 3 years of age (the most critical age for agenesis of a tooth) at the time of the accident should be performed.

Gingival pigmentation was observed in two (Caucasian) subjects from the ABR zones and in two from the non-ABR zone. In all subjects the pigmentation was mild and may be related to factors other than TCDD exposure. This is in contrast to the findings in Yusho patients. Oral pigmentation was observed in 75 of 121 patients approximately 30 years after the accident, with gingival pigmentation predominating (Hashiguchi et al. 2003). This striking difference is difficult to explain but may be partly related to differences in the properties of the causative compounds.

Prominent and confirmed risk factors or predictors of periodontal diseases in adults include smoking and low education (Pihlstrom 2001). In our study the subjects were frequency matched with education, and smoking was as common in subjects from the ABR zones (35%) as in subjects from the non-ABR zone (33%). Because we found no significant associations between serum TCDD levels and the periodontal disease parameters and no differences in the prevalence of periodontal disease among subjects from the ABR zones and the non-ABR zone, we suggest that exposure to TCDD in childhood is not associated with the development of periodontal disease.

In Yusho patients periodontal disease was a frequent finding (Hashiguchi et al. 2003). About 30 years after the accident, 95 of 110 examined patients (86%) had at least one tooth with a periodontal pocket deeper than 3 mm. Unfortunately, no information on the controls was available. The authors suggested that PCBs and related compounds might play an important role in the development of periodontal disease (Hashiguchi et al. 2003). Such a tendency was not seen in our study population exposed mainly to TCDD.

In a case report, Shimizu et al. (1992) described dento-orofacial characteristics of a 24-year-old female who had been exposed to PCBs and PCDFs at 6 years of age. Her main clinical findings were oral hyperpigmentation, periodontitis, and delayed eruption of teeth. Radiologic findings were a cystic radiolucency in the mandible and hypoplastic and dilacerated roots in developing teeth. Some teeth were impacted, malposed, and ankylosed. Distortion of the roots has also been reported in Yusho patients by Fukuyama et al. (1979). Unexpectedly, in the present study radiographic examination did not reveal developmental cysts, root dilacerations, or impacted teeth (except for an upper canine in one subject). Nor did the dental histories reveal remarkable aberrations.

Our recent findings show that TCDD impairs branching morphogenesis of mouse embryonic salivary glands *in vitro* (Kiukkonen A et al., unpublished data). Given our present results, it seems that the function of salivary glands measured as salivary flow rate was not affected in subjects exposed to high amounts of TCDD in Seveso.

Taken together, these results from Seveso show that developmental dental aberrations, which are permanent in nature, were related to childhood exposure to TCDD. In contrast, dental caries or periodontal diseases, which are infectious in nature, were not associated with the exposure. The results support our hypothesis that dioxins can interfere with human organogenesis.

## Figures and Tables

**Table 1:** Characteristics of the study population.

Variable	Zone ABR	Zone non-ABR	*p*-Value
Total	48	65	
Sex (female)	23 (48)	37 (57)	0.34
Age			
Mean (range), years	29.1 (25.4–34.0)	29.2 (24.6–34.1)	0.93
< 5 years at the time of the accident	36 (75)	39 (60)	0.095
Education			
Elementary school	2 (4)	5 (8)	
Secondary school not finished	6 (12)	7 (11)	
Vocational school	11 (23)	6 (9)	
Secondary school finished	23 (48)	34 (52)	
High school finished, university training	6 (12)	13 (20)	0.28
Smoker	15 (31)	21 (32)	0.90

Values shown are number (%) except where noted.

**Table 2:** Presence of developmental defects of enamel by age and by zone of residence at the time of the accident, serum TCDD level, and education in children < 5 years of age at the time of the accident.

Variable	No. of subjects	No. of subjects with developmental defects of enamel (%)	*p*-Value
> 5 years of age at time of accident	38	2 (5.3)	
< 5 years of age at time of accident	75	25 (33)	0.0009
Non-ABR zone	39	10 (26)	
ABR zone	36	15 (42)	0.14
31–226 ng/kg TCDD	10	1 (10)	
238–592 ng/kg TCDD	11	5 (45)	
700–26,000 ng/kg TCDD	15	9 (60)	0.016
Educational level			
ABR zone			
Lower*^a^*	6	5 (83)	
Higher*^b^*	30	10 (33)	0.023
Non-ABR zone			
Lower	6	3 (50)	
Higher	33	6 (18)	0.14

a Lower education level refers to subjects with elementary school or secondary school not finished (basic education).

b Higher education level refers to subjects with vocational school, secondary school finished, high school finished, or university training.

**Table 3:** Independent explanatory factors associated in the logistic regression model with the presence of developmental enamel defects in subjects < 5 years of age at the time of the Seveso accident.

Variable	*p*-Value	OR (95% CI)
TCDD exposure level	0.007	
Resident from non-ABR zone or serum TCDD 31–226 ng/kg in serum lipid		1.0
Increase of the serum TCDD level (238–592 and 700–26,000 ng/kg in serum lipid)		2.4 (1.3–4.5)
Education	0.014	
Higher than basic education		1.0
Basic education only		5.8 (1.4–23.7)

Abbreviations: CI, confidence interval; OR, odds ratio. Residents from zone non-ABR and those with the lowest tertile of the ranked serum TCDD concentrations from zones ABR are grouped.

**Table 4:** Dental caries, periodontal disease, and salivary flow in subjects from ABR zones (*n* = 48) and from the non-ABR zone (*n* = 65) [mean ± SD (range)].

Variable	Zone ABR*^a^*	Zone non-ABR
DMFT	11.7 ± 4.05 (3–21)	10.6 ± 5.26 (0–22)
Attachment loss at probing > 4 mm (no. of sites)	7.7 ± 6.8 (0–28)	7.2 ± 8.6 (0–33)
Attachment loss at probing > 5 mm (no. of sites)	1.3 ± 2.2 (0–10)	1.7 ± 4.5 (0–31)
Bleeding on probing (percent of sites)	33.9 ± 16.0 (6–63)	31.0 ± 15.6 (3–71)
Subgingival calculus (percent of sites)	29.1 ± 14.4 (1–61)	27.0 ± 16.2 (1–77)
Stimulated salivary flow rate (mL/min)	1.3 ± 0.64 (0.4–2.4)	1.3 ± 0.75 (0–3.6)

a The difference between the mean values of all variables of zone ABR subjects and zone non-ABR subjects was insignificant (range of *p*-values, 0.11–0.67).
